# Primary healthcare system and practice characteristics in Singapore

**DOI:** 10.1186/s12930-014-0008-x

**Published:** 2014-07-19

**Authors:** Hwee Sing Khoo, Yee Wei Lim, Hubertus JM Vrijhoef

**Affiliations:** 1 Saw Swee Hock School of Public Health, National University of Singapore, MD3, 16 Medical Drive, Singapore 117597, Singapore; 2 RAND Corporation, Santa Monica, California; 3 Scientific Centre for Care and Welfare (Tranzo), Tilburg University, Tilburg, The Netherlands; 4 National Healthcare Group, Health Outcomes and Medical Education Research (HOMER), Singapore, Singapore

**Keywords:** Primary care, Practice characteristics, System characteristics, Quality, Singapore

## Abstract

It is crucial to adapt and improve the (primary) health care systems of countries to prepare for future patient profiles and their related needs. The main aim of this study was to acquire a comprehensive overview of the perceptions of primary care experts in Singapore about the state of primary care in Singapore, and to compare this with the state of primary care in other countries. Notwithstanding ranked 2^nd^ in terms of efficiency of health care, Singapore is facing significant health care challenges. Emails were sent to 85 experts, where they were asked to rate Singapore’s primary care system based on nine internationally adopted health system characteristics and six practice characteristics (response rate = 29%). The primary care system in Singapore received an average of 10.9 out of 30 possible points. Lowest ratings were given to: earnings of primary care physicians compared to specialists, requirement for 24 hr accessibility of primary care services, standard of family medicine in academic departments, reflection of community served by practices in patient lists, and the access to specialists without needing to be referred by primary care physicians. Singapore was categorized as a ‘low’ primary care country according to the experts.

## Background

All health care systems across the world include primary care. However, the level of development of primary care as part of the health care system varies substantially. In strengthening primary care, lessons can be learned from other countries. This has become increasingly important as various countries are instituting policies to hold primary care practices accountable for managing chronic conditions and meeting clinical standards. Also in Singapore this is the case, the country ranked second amongst countries with most efficient health care [1].

Traditionally, health care systems of countries focused on acute, episodic care, addressing the needs of inpatients. Many are now moving towards holistic care, to a health care system that takes into consideration the ageing population and the corresponding increase in chronic diseases [2]–[4]. Moreover, an ageing population would need regular care that should be available not only in acute hospitals, but in the community. However, the ageing population and inevitable rising health care costs in Singapore suggests the necessity for an assessment of its current primary care system as a strong primary system could reduce cost and contribute to improvements in health [5]. In this paper, we sought the opinions of primary care experts in Singapore where they rated the primary care system of the country and to compare this with the state of primary care in other countries.

In general, the primary care system should and has to become the mainstay in the long term management of patients, such as those with diabetes, heart failure or chronic lung diseases. Primary care can be defined as “that level of a health service system that provides entry into the system for all new needs and problems, provides person focused care over time, provides care for all but very uncommon or unusual conditions, and co-ordinates or integrates care provided elsewhere or by others” [6]. Primary health care would encompass primary medical treatment services, as well as education on preventive health care and health. However, it is not possible to create effective primary care systems using a “one size fits all” approach, or put into practice one recipe, as systems are dependent on context. The development of a primary care system would be shaped not only by the health problems the country faces, but also the country’s historical background, and societal beliefs and values. The strength of a country’s primary care system is hence reliant on how well the above primary care dimensions develop within the context of a country’s health care system [7].

The increasing population, coupled with an ageing population, is a combined challenge for Singapore. The population has grown 25% over the past decade and will continue to grow. It is estimated that 20% of Singaporean residents will be aged 65 and above by year 2030. By year 2100, Singapore is projected to have a median age of 56.4 years, the highest of world population prospects. Comparatively, Japan has the highest median age of 45.9 in 2013, and is projected to have a median age of 51.8 in 2100, ranking 8^th^ highest in the world [8]. This suggests an increased and urgent demand for health care in the future for Singapore as the elderly require more medical care. Other than longer hospital stays, chronic diseases that require long term management from health care professionals also affect the elderly [9].

It is crucial to continue to adapt and improve the (primary) health care system in Singapore to prepare for future patient profiles and their related needs. It would be more effective and sustainable to manage the chronic conditions of the elderly in the community than in acute hospitals in the long run. However, continuity of care appears to be low in Singapore, with only 38.4% of residents indicating in a national health surveillance survey that they go to a regular family doctor [10]. The main reason that patients seek treatment at the government run and subsidized polyclinic is for chronic diseases [11]. This suggests that the costs of services could be a factor for patients choosing subsidized services, since chronic disease management requires follow-up consultations. The rise in health care costs is another area that has to be monitored to maintain cost-effectiveness, so that health care may stay affordable for everyone [12]. Therefore it is of utmost relevance to assess the current state of the primary health care system and practice characteristics in Singapore to see if and how it can be improved.

The main aim of this study was to acquire a quick and comprehensive overview of the perceptions of primary care experts in Singapore about the standard of primary care in Singapore, and to compare this with the state in other countries. This study is the first to assess the overall strengths, weaknesses, and characteristics of the primary health care model as part of the Singapore health care system. The results may be used as a basis for comparison with other Asian countries and to inform future health care reforms and research.

### Gap between theory and practice

A gap still remains between conceptual models of care and existing provider practice despite a focus on improving primary health care systems to cope with the increasing needs. It is however challenging to sustain programs promoting integrated services to optimize resource utilization partly due to the difficulty in acquiring the involvement and participation of health care professionals in the private sector, for instance, general practitioners (GPs). Interviews conducted by researchers in the United States with GPs revealed it is difficult to care for elderly patients in practice environments that do not provide the support and resources required by such patients. The difficulty in caring for elderly patients was due to three main issues: 1) medical complexity and chronicity as elderly patients are more vulnerable to quick declines in their health conditions, 2) personal and interpersonal challenges, and 3) increased administrative burden [13]. Indeed, this reflects a lacuna that should be filled, and highlights the need to provide sufficient support to the primary care sector if their increased participation in caring for the elderly is viewed as a desired outcome in the future.

In Singapore, primary health care is provided by government polyclinics and private general medical practitioner clinics. These health care professionals are usually the first point of contact with patients. The eighteen polyclinics provide about 20% of primary health care; while around 2,000 private medical clinics provide the remaining 80%. There has however been an imbalance in the share of chronic disease management in the primary care sector in Singapore. Even though private general practitioners currently provide around 80% of primary care in Singapore, only 55% of chronic patients are managed by them, while polyclinics cope with the remaining 45% of chronically ill patients [14].

For primary care in Singapore, a complete range of medical care for both acute and chronic medical conditions are provided by the polyclinics, including medical facilities and comprehensive health care services including outpatient medical care, health screening, education, and vaccinations, and x-ray and laboratory services, allowing them to be a one-stop health centre for the community [15]. Private clinics could be made up of solo, small group or large health care group practices. These private clinics usually do not possess onsite investigative facilities and are not subsidized by the government, unlike the eighteen polyclinics. Secondary and tertiary care is provided by the eight national specialty centers and seven acute public hospitals. As of 2010, there are 8,797 doctors active in Singapore: 5,362 in the public sector and 3,435 in private practice [16].

The main difference between the polyclinics and private clinics is that patients are assigned any doctor from a common group of medical officers and family physicians, while there is usually one main family physician at private clinics, ensuring a higher possibility of continuity of care. Polyclinic patients may also choose to see the doctors from the Family Physician Clinic in the polyclinic which ensures them care continuity from the same doctor, at a higher rate, but still considerably subsidized [17],[18]. While polyclinics are highly subsidized by the government, there is also a heavy patient load where the polyclinic doctors see 58 patients each day, generally resulting in a much longer waiting time for consultation, compared to 30 patients per day for a private clinic family physician [19]. GPs worked an average of 52.5 hours a week (7.5 hours a day) [20]. Despite the long waiting time and low care continuity, chronic disease patients sought treatment mainly from government polyclinics, burdening the limited subsidized resources of the polyclinics. This is mainly due to the higher cost of care at GP clinics as compared to subsidized treatments and medication at polyclinics [21].

### Health care financing in Singapore

Singapore adopts a mixed health financing system that emphasizes individual responsibility and an attempt to avoid moral hazards that could be faced with pure national insurance schemes when health care is provided for free. Health care is funded jointly by the government and the individual through insurance, revenue from taxes, as well as savings from each individual’s medical savings account (i.e. Medisave) [22]. To ensure that basic medical care is accessible to everyone, public hospitals, polyclinics, as well as nursing homes are directly subsidized by the government (up to 80% of the total bill in acute public hospital wards) [23].

Singapore’s health care outcomes are comparable to other developed nations, considering that around 4% of Singapore’s Gross Domestic Product (GDP) is spent annually on health care as compared to the United States (17.9% of GDP) and the United Kingdom (9.6% of GDP) [24]. Singapore’s life expectancy from birth in 2011 is currently 82 years for both sexes, compared to the regional average of 75 years, and the global average of 68 years old [25].

At the polyclinics, Singapore citizens less than 18 years of age and above 65 receive up to 75% concessions in consultation and treatment fees, while all other Singapore citizens are given a 50% concession for their fees.

## Methods

Our study made use of the primary care framework by Starfield [26]. The framework has been administered in many countries including the United Kingdom, Denmark, The Netherlands, Japan, Australia, Sweden, the United States, Austria, and Germany [27]. Starfield’s framework of primary care encompasses both health system and practice characteristics [26]. The primary care framework has been applied in the different countries with varying primary care systems. These characteristics contribute to the strength of primary care in countries. The nine *health system characteristics* include: 1) how much the distribution of resources throughout the country is controlled by the system, 2) how primary care services are financed, 3) the main type of primary care practitioner in the country - a higher percentage of generalists would receive a higher score, 4) the percentage of physicians in primary care as compared to specialty care, 5) the ratio of professional earnings of primary care physicians in contrast to specialists, 6) the extent of cost shared by patients, 7) reflection of community served by practices in patient lists, 8) 24 hr accessibility of primary care services, and 9) the academic strength of primary care or general practice departments. The six *practice characteristics* include: 1) first contact care (where a patient needs to be referred to a specialist through a primary care physician), 2) longitudinality (person-focused care over time), 3) comprehensiveness of care, 4) coordination of care, 5) family-centeredness or care, and 6) community orientation (practitioners use community data to plan or organize services, or identify problems).

Following Starfield and Lei’s [28] paper, the information on the nine health system characteristics and six practice characteristics were acquired from primary care system experts who have published in peer-reviewed journals on the primary care system in Singapore and/or general practitioners with more than 10 years of experience. The primary healthcare experts were selected from both private and public sectors, where 32% are female.

Emails were sent to 85 experts where 25 responses were received, a 29% response rate. Respondents were allowed to mail hardcopy responses to the authors to maintain their anonymity. As of 31 March 2012, there are 1572 registered members in the College of Family Physicians Singapore [29].

We corresponded individually with these experts through email to acquire ratings that were independent from each other. A standardized email was sent to all the experts with step-by-step instructions regarding the rating process. For each characteristic, comprehensive and explicit criteria were included on when the expert should assign 2, 1, or 0 points. A characteristic is rated with a score of 2 for ‘high’ level of development, 1 for ‘moderate’ level of development, or 0 for ‘absence or low’ level of development. The most frequently assigned score was selected as the final score to achieve inter-rater agreement [6]. Further, the average sum score was calculated by dividing the total sum scores by the number of respondents.

Descriptive statistics were used to calculate overall scores using Microsoft Office Excel 2007. Approval was attained from the Institutional Review Board of National University Hospital System for our study.

## Results

For all but one item, the majority of experts rated items having low level of development (6 items) or modest level of development (5 items) and three items were rated of low or modest level by an equal number of respondents. Lowest ratings were given to: earnings of primary care physicians as compared to specialists, requirement for 24 hr accessibility of primary care services, standard of family medicine in academic departments, reflection of community served by practices in patient lists, and “first contact” (the need to be referred to a specialist by the primary care physician). Most experts rated main type of primary care practitioner the highest, where the high score of 2 is given if the main type of primary care practitioner in the country are generalists, while the low score of 0 is given if the main type of primary care practitioners in the country do not focus solely on family medicine. Table 1 presents the frequency of scores by the experts. Table 2 displays the health care system and primary care practice characteristics, along with an abbreviated description of the characteristics, followed by the final score. The descriptions of the characteristics were derived from literature about Singapore’s health care system as well as comments from the experts.

**Table 1:** Frequency of scores by experts

Item#	1	2	3	4	5	6	7	8	9	10	11	12	13	14	15
2 points	3	4	13	8	2	1	0	0	1	3	1	7	1	2	3
1 point	14	15	11	15	3	**12**	6	9	8	10	**12**	16	**12**	23	8
0 points	8	6	1	2	20	**12**	19	16	16	12	**12**	2	**12**	0	14
Final score (mode)	1	1	2	1	0	-	0	0	0	0	-	1	-	1	0
Sum score (Item x point)	20	23	37	31	7	14	6	9	10	16	13	30	14	27	14
Average (sum score/25)	0.8	0.92	1.48	1.24	0.28	0.56	0.24	0.36	0.4	0.64	0.56	1.2	0.56	1.08	0.56
Total sum score	10.9														

**Table 2:** Primary care health and system characteristics scores

**Item number**	**Characteristics**	**Description of characteristic**	**Final score**
Health care system characteristics			
1	Type of system	Polyclinic locations are regulated by the government to provide sufficient care around Singapore	1
2	Financing	Partly tax based. A combination of government subsidies, an individual compulsory medical savings account, and a low cost insurance scheme	1
3	Type of practitioner	GPs in the country are mainly generalists focusing on family medicine, and not specialists in other disciplines.	2
4	Percentage who are specialists	38.77% of doctors in Singapore are specialists [16], indicative of an orientation toward primary care	1
5	Primary care physicians earnings compared to specialists	Specialists earn more than primary care physicians	0
6	Cost sharing	A combination of government subsidies, an individual compulsory medical savings account, and a low cost insurance scheme	-
7	Patient Lists	There is no requirement to sign up with a personal GP.	0
8	Requirement for 24-hour coverage	No regulated requirement for 24 hour primary healthcare. Patients may visit 24 hr A&E (accident and emergency) departments when necessary.	0
9	Standard of family medicine academic departments	Family medicine in Singapore is given low priority.	0
Practice characteristics			
10	First contact	Patients may choose to be referred by a primary care physician or choose to go to a private specialist directly.	0
11	Longitudinality	Patients do not get to select their doctors when they visit a polyclinic, and there is no system to enroll patients (patient lists) for private general practitioners.	-
12	Comprehensiveness	Polyclinics and private group GPs have a comprehensive range of services and facilities. Community Health Centres provide off-site ancillary support services to GPs without full facilities.	1
13	Coordination	Poor coordination and information transfer between primary, secondary and tertiary levels of healthcare	-
14	Family-centeredness	Family members are informed of medical decisions in hospitals	1
15	Community orientation	Data from practitioners not analyzed or used to identify priorities of care for the community	0

Singapore received an average of 10.9 out of 30 possible points (271/25). Nine characteristics were rated as ‘0’ and one characteristic received a high rating of 2 points. The health system characteristics were rated an average of 6.3 out of 18 possible points by the experts (157/25). Under the “type of practitioner” system characteristic, generalists are the main type of primary care practitioners in Singapore, and this characteristic received a high rating of “2” from the experts. The practice characteristics were rated an average of 4.6 out of 12 possible points by the experts (114/25). The practice characteristics of “first contact” and “community orientation” both received low scores of “0” from the experts. First contact implies that decisions about the need for specialty services are made after consulting the primary care physician. Requirements for access to specialists via referral from primary care are considered most consistent with the first-contact aspect of primary care. The ability of patient to self-refer to specialists is considered conducive to a speciality-oriented health system, and is rated low. Community orientation describes whether the doctor actively seeks to understand important health problems in the neighborhood. Low ratings are assigned when there is little or no attempt to use data from the practice to plan or organize services and identify priorities for care.

## Discussion

The results of our preliminary study suggest that improvements could be made to the primary care system in Singapore, and much more work needs to be done. Based on the average sum score (10.9, over a total of 30 possible points), Singapore can overall be considered a ‘low primary care country’ when compared with other countries that utilized the same assessment tool [28]. See Table 3[28] comparing Singapore with other countries [28].

**Table 3:** Primary care scores

**Country**	**System score (Characteristics 1–9)**	**Practice score (Characteristics 10–15)**	**Total score (max. 30)**	**Total average score (max. 2)**
*Low primary care*				
Belgium	5.6	0.0	5.6	0.4
France	5.0	0.0	5.0	0.3
Germany	6.0	0.0	6.0	0.4
United States	4.0	1.5	5.5	0.4
*Intermediate primary care*				
Australia	10.0	7.0	17.0	1.1
Canada	11.5	6.0	17.5	1.2
Japan	8.5	4.0	12.5	0.8
Sweden	10.0	4.0	14.0	0.9
*High primary care*				
Denmark	16.0	10.0	26.0	1.7
Finland	15.0	7.0	22.0	1.5
The Netherlands	13.0	10.0	23.0	1.5
Spain	12.5	8.0	20.5	1.4
United Kingdom	18.0	11.0	29.0	1.9

*Scores in Table*3*are obtained from Starfield & Shi*[28].

Among the 15 dimensions measured, six had scores of zero. These dimensions included measures that point to the status of primary care compared to specialist care, accessibility of care (24 hrs), as well as measures of access and care continuity. The latter group of measures are particularly pertinent to the care of patients with chronic and complex conditions. The inevitable increase in the elderly population with chronic diseases will place pressure on the health care system. If the primary care system, as reflected by the low scores in care continuity, does not develop in the areas of care continuity, care for the elder patients with chronic diseases will likely be suboptimal.

Regarding the low score of 24 hr accessibility of care in Singapore, the US and Canada similarly do not have policies for after-hours coverage [30]. In a 2012 international survey of primary care doctors conducted by the Commonwealth Fund, only 34 percent of US practices have arrangements for their patients to see their doctors or nurses without going to a hospital emergency department. This is in contrast to countries such as the Netherlands, New Zealand, and the UK, where more than 90 percent of physicians reported having after-hours coverage in place [31]. The low percentage reported by Canadian and US physicians suggest that after-hours coverage develops slowly if it depends on solo practices, [32] as compared to requirements such as national help lines in the UK, and payment incentives to physicians to provide after-hours care in Australia.

Care coordination is a major challenge for primary care systems, especially with the increase in the elderly population and the attention and long term management required for chronic diseases that are commonly afflict the elderly. While this dimension received a tied score between 0 and 1 from the primary care experts in Singapore, the 2012 Commonwealth Fund survey revealed that care is generally not well coordinated in the ten countries surveyed. Besides physicians from France and Switzerland, where more than 50 percent said that a report with related health information will be sent to them after their patients saw a specialist, less than 20 percent of physicians in the US, the Netherlands, and Germany reported that they received a relevant health report from the specialist [31].

In Singapore, there is no patient list system and patients have the freedom of selecting any doctor they wish to see. Patients therefore have direct access to specialist care. Such health systems are described as more expensive as compared to a system where patients have to be referred through the primary care system [33]. This is also related to the practice characteristic of “first access”, where the primary care system is rated well if patients need to be referred by primary care physicians to a specialist. Looking at this from the perspective of health care finance, the variation in our findings as well as the comments provided by the experts indicate that there is not a consistent judgement on what might be the right model of health care finance in Singapore. In a sense, Singapore has shown that it is possible to achieve an efficient health care system at lower cost. In terms of Starfield’s model, the “financing” health system characteristic could be revised to accommodate more permutations of health care financing. Different financing combinations might be more suitable for different countries based on every country’s unique characteristics. The health care system of Singapore is currently in transition, [34] and in line with this, the Ministry of Health recently presented a ‘Health care 2020’ Masterplan comprising of a set of strategies to guide Singapore towards an inclusive health care system for the future [35]. The aim is to enhance accessibility, quality, and affordability of health care for all people in Singapore, and the further development of primary care is a part of this strategy. Besides the plan to increase subsidies from the government for chronic outpatient treatment at GPs for low-income and middle-income families, most significantly, three new primary care models will get developed: Family Medicine Clinics (FMCs), Community Health Centres (CHCs) and Medical Centres (MCs). The different models are meant to be all encompassing to cater to patients with different profiles and preferences. This is a step towards improving system and practice characteristics such as “patient lists”, “coordination”, “comprehensiveness”, and “family centeredness”. It is expected that with these models more patients can be cared for outside the hospitals’ specialist outpatient clinics by GPs in the community.

One example of the FMC initiative would be Frontier FMC (a private GP clinic) in Singapore. In collaboration with the National University Health System (NUHS), it is applying the Patient Centered Medical Home concept (PCMH) [36] in Singapore, based on AAFP’s (American Academy of Family Physicians) joint principles of PCMH [37]. It is expected that patients with chronic conditions (i.e. diabetes, hypertension, stroke and asthma) who no longer require specialist care can enjoy shorter waiting time when they seek outpatient treatment and follow up at the Frontier FMC in the community. Frontier FMC was opened in the first week of April 2013 [38]. Besides encouraging information transfer between GPs and specialists, at the same time, through team based care, it is expected to slow down disease progression, reduce complication rates and in turn, minimize referrals to hospitals. Since it is the first implementation of the PCMH in Singapore it is yet unclear *how* the proposed solutions to improve chronic illness care will play out. Important components of the new approach include: team based care, electronic health record shared between NUHS and Frontier FMC, and a referral system for those with complex care needs.

Finally, another two characteristics that scored zero (primary care physician earnings as compared to specialist, and family medicine as academic department) indicate the need to raise the profile of family medicine and the role primary care physicians play in overall health care delivery. Even with an optimal health care finance mechanism and an integrated care system in place, the relatively low status of primary care education and research will impede the development of a stronger primary care delivery system in Singapore.

There are a couple of limitations we should note for this study. First, the response rate to our study was relatively small. Despite the relatively small response, the variability in scores between respondents suggests that the study group varies in their opinions about primary care as one would expect (from previous studies in other countries). Second, Singapore’s scores could not be compared directly with the scores from other countries as there were differences in opinions about the characteristics, unlike the scores attained by Starfield and Shi [28] where it was noted that informants had “no disagreements” in the scoring of the characteristics. This was also the reason we decided to use average sum scores instead of relying on the modal score. Our scores for both system and practice characteristics were also attained primarily from experts in primary care in Singapore, while the system scores in Starfield & Shi [28] were mainly obtained from the Organization for Economic Cooperation and Development (OECD) [39].

The assessment of health care quality, especially regarding chronic diseases, has more often been based on the perspectives of the care provider or the health care institution than the experience of the patient [40]. The terms patient perception and patient satisfaction have often been used in place of each other, [41] but it should be noted that satisfaction is one way of describing and not the only illustration of a perception. Despite this, patient satisfaction has been a main feature in quality care assessment studies. Annual patient satisfaction surveys have been conducted in Singapore, but these surveys are usually unable to capture the full spectrum primary care dimensions related to patient satisfaction. These surveys provide only a partial picture of the quality of health care. It is essential to attain the patient perspective to understand the patient experience in terms of the reasons for patient preferences, as well as the obstacles in the current primary health care model encountered by patients while seeking primary health care to identify an accurate and fairer perception of which characteristics should be focused upon to improve the primary health care system. This would allow a clearer picture of how the current primary care system is working, than if perspectives were only attained from the primary care experts and physicians’ points of view.

## Conclusion

In all, this study is the first to document the standard of primary care in the Singapore health care system from the perspective of primary care experts. Systematically listening to doctors on the front lines of primary care can help identify gaps and target reforms of health systems. According to primary care experts from Singapore, the strength of its primary care system is low. This is an important concern considering the developments in its population. The study provided us with a comprehensive overview of the standard of primary care in Singapore as perceived by primary care experts in Singapore, and also allowed us to compare the state of primary care in Singapore with other countries. The results can be used as a basis to inform future health care reforms and research, for comparison with other (East) Asian countries, and to assess trends in time if the survey would be applied again in the future.

## Competing interests

The authors declare that they have no competing interests.

## Authors’ contributions

KHS contributed in the conception and design of the survey, data collection, drafting the manuscript and making critical revisions. LYW contributed in the conception and design, data collection and critical revisions. H Vrijhoef contributed in conception and design, and critical revisions. All authors have given final approval of the version to be published and agree to be accountable for all aspects of the work.
